# Enhancing Engagement With Endocrine Guidelines and Fostering Medical Student Interest Through Concise Medical Information Cines: Qualitative Co-Design Study

**DOI:** 10.2196/83711

**Published:** 2026-01-27

**Authors:** Rahul Sagu, Sangamithra Ravi, Aspasia Manta, Punith Kempegowda

**Affiliations:** 1 Birmingham Medical School, College of Medicine and Health, University of Birmingham, Birmingham, United Kingdom; 2 Department of Applied Health Sciences, School of Health Sciences, College of Medicine and Health, University of Birmingham, Birmingham, United Kingdom; 3 Department of Endocrinology and Diabetes, Queen Elizabeth Hospital Birmingham, University Hospitals Birmingham NHS Foundation Trust, Mindelsohn Way, Birmingham, B15 2GW, United Kingdom, 44 7721930777

**Keywords:** guidelines, education, endocrinology, dissemination, implementation

## Abstract

**Background:**

There is a need to modernize the dissemination of clinical guidelines, making them more accessible and engaging for health care professionals. Concise Medical Information Cines (CoMICs) are peer-reviewed videos created by medical students that distill complex guidelines into learner-friendly visuals.

**Objective:**

This study aimed to describe the process of co-designing an audiovisual version of a clinical guideline and explore the experiences of co-designing audiovisual guideline summaries using the CoMICs model.

**Methods:**

A 4-part CoMICs series on glucocorticoid-induced adrenal insufficiency was codeveloped by clinicians and medical students through 10 iterative steps. A patient version of these CoMICs was then created in multiple languages. Semistructured interviews with authors, reviewers, and student collaborators assessed the clarity, usability, trustworthiness, and educational value of these CoMICs. Reflexive thematic analysis then identified key themes.

**Results:**

CoMICs improved guideline accessibility, comprehension, and global adaptability, while the collaborative process promoted interdisciplinary learning and underscored the efficacy of audiovisual tools for complex content. Student collaborators reported greater confidence in interpreting and communicating clinical guidance, renewed interest in endocrinology, and a deeper appreciation of its academic dimensions.

**Conclusions:**

Cocreating audiovisual resources, such as CoMICs, enhances guideline dissemination. Student involvement can foster curiosity, encourage academic career pathways, and reshape engagement with evidence-based medicine.

## Introduction

Clinical practice guidelines are a cornerstone of evidence-based medicine, offering systematically developed recommendations to optimize patient care [1]. Despite their importance, ensuring that guidelines are effectively disseminated and implemented in clinical settings remains a persistent challenge. Health care professionals often face barriers such as time constraints, cognitive overload, lack of awareness of updates, and perceived limited contextual relevance—factors that reduce the usability of traditional long-form, text-based documents in fast-paced environments [2].

Dissemination refers to the targeted distribution of evidence-based knowledge and materials to specific audiences to increase awareness and understanding [3]. Conventional dissemination approaches, such as printed summaries, educational events, audit and feedback loops, and reminder systems, have shown variable success [4]. While multifaceted interventions tend to outperform single strategies, their implementation is often constrained by organizational resources, and they fail to achieve consistent improvements in clinical behaviors or patient outcomes. Implementation, in this context, involves systematically integrating that knowledge into real-world practice to improve health care outcomes. More importantly, these strategies frequently overlook the importance of engagement, accessibility, and real-time utility—features that are increasingly essential in today’s diverse and dynamic health care systems [5]. These limitations underscore the need for supplementary dissemination strategies that can deliver key recommendations in more accessible, engaging, and time-efficient formats [1].

Emerging audiovisual tools offer an opportunity to bridge this gap. By reducing cognitive load, enhancing recall, and improving user engagement, visual formats can complement traditional learning formats [6]. One such approach is Concise Medical Information Cines (CoMICs)—short, peer-reviewed, visually engaging videos designed to translate complex clinical guidance into digestible content [7]. Using infographics, narration, and visual storytelling, CoMICs aim to make guidelines more approachable and usable for both early-career and experienced professionals.

In parallel with growing calls to diversify how students experience specialty fields and engage with research, coproducing educational resources such as CoMICs allows students to gain exposure to academic medicine in a hands-on, creative manner. The Endocrine Society’s Medical School Engagement Program similarly seeks to broaden medical students’ exposure to endocrinology [8]. By aligning with the goals of the Medical School Engagement Program, CoMICs offer a complementary, hands-on platform that both disseminates core guideline content and actively engages students in the specialty. While the earlier CoMICs study investigated the dissemination and engagement patterns of specialty-focused educational videos on social media platforms, the translation of a clinical guideline into a structured audiovisual format has not been examined in previous CoMICs research. Therefore, in this study, we describe the process of codeveloping a 4-part CoMICs series based on the glucocorticoid-induced adrenal insufficiency (GIAI) guideline, showcasing an innovative approach to translating clinical guidance into accessible resources. We also explored medical students’ experiences of co-designing the CoMICs and how this influenced their learning.

## Methods

### Overview

This study was conducted between October 2024 and May 2025 at the University of Birmingham. The process of CoMICs creation, aligned with the knowledge-to-action framework, has been described in detail in our previous publication [7]. To inform the adoption of CoMICs for guideline dissemination, we conducted a structured exploratory literature review using PubMed. The search strategy focused on 3 intersecting themes: dissemination methods, barriers to the uptake of clinical guidelines, and health care professional behaviors. The review was limited to English-language articles involving health care professionals but included studies from all publication years to ensure comprehensive coverage. Key findings highlighted the limitations of traditional dissemination techniques, such as printed materials, educational sessions, and audit-feedback cycles, and underscored the need for more engaging and accessible formats, particularly in time-pressured clinical environments [4]. These insights shaped the rationale for the CoMICs approach. A 4-part audiovisual series was created to convey the core content of the Joint European Society of Endocrinology and Endocrine Society guideline on GIAI. The primary focus of this work is qualitative, centered on the co-design and evaluation of CoMICs. The literature review served as a supporting framework for this study. Each video distilled key diagnostic, therapeutic, and follow-up recommendations into concise, visually engaging episodes. Crucially, medical students played an active role throughout the ten-step CoMICs development process, which included the following:

1. The CoMICs team contacted the corresponding guideline author to establish collaboration, define the scope of the project, and confirm participation in an iterative review.
2. Guideline authors shared supplementary educational resources (eg, slide decks, figures, explanatory notes, or key references) to support accurate interpretation of the guideline.
3. The CoMICs team developed an initial script and visual storyboard using PowerPoint (Microsoft Corp), translating key guideline recommendations into simplified, learner-friendly visual narratives.
4. The storyboard underwent clinical review by guideline authors, who provided feedback on accuracy, emphasis, and clarity, after which suggested revisions were incorporated.
5. A preliminary video draft without narration or background audio was produced to enable focused review of structure, visuals, and content flow.
6. Feedback from guideline authors, clinical experts, and peer reviewers was collated by the CoMICs team and used to refine content, visuals, and sequencing.
7. A prefinal version incorporating narration and refined visuals was produced and circulated to the authors for final clinical validation.
8. Final comments and minor revisions were addressed to ensure consistency with the guideline and clarity for the intended audience.
9. The completed CoMIC was approved by the corresponding author and prepared for professional use.
10. Approved videos were disseminated through clinical networks and social media platforms to maximize accessibility, reach, and engagement [9].

This process not only supported the technical production of CoMICs but also served as a structured educational opportunity, exposing students to academic publishing, clinical content curation, and interdisciplinary teamwork in endocrinology. The CoMICs videos were disseminated through multiple online platforms to maximize reach and engagement. All videos were uploaded to YouTube (Google LLC) as the primary hosting platform and shared via social media channels, including X (X Corp, formerly Twitter), Instagram (Meta Platforms, Inc), and WhatsApp (Meta Platforms, Inc) groups within the CoMICs network. The posts were accompanied by short educational captions and reposted by CoMICs members on the aforementioned platforms to enhance discoverability.

### Multilingual Patient-Focused CoMICs Development

In parallel, a patient-facing version of the CoMICs series was developed to improve health literacy and access among lay audiences. These 4 short videos were co-designed with individuals living with adrenal insufficiency and deliberately avoided technical language. Patient advocates provided feedback throughout script development, ensuring clarity, relevance, and the inclusion of relatable, everyday examples. Practical guidance on symptom recognition, treatment adherence, and self-management was integrated.

To further enhance accessibility, multilingual versions were created. Medical students fluent in English and another language of interest to this project produced initial transcripts, which were then reviewed by native speakers with clinical or health communication experience. This ensured that idiomatic clarity, cultural appropriateness, and medical accuracy were preserved across language versions. Languages for the multilingual video adaptations were selected pragmatically based on the connections within the CoMICs network and the availability of fluent volunteer translators. This approach ensured linguistic accuracy while allowing culturally relevant adaptations for each audience. This process enhanced the reach and relevance of the CoMICs series to non–English-speaking audiences, promoting global health equity [10].

### Qualitative Interviews to Explore Experience and Impact

Purposive sampling was used to recruit participants with direct involvement in the CoMICs project or relevant expertise. These included the following:

- Medical students and health care professionals involved in the CoMICs cocreation
- Clinical experts who authored or peer-reviewed the GIAI guideline

Among the CoMICs team members interviewed, there were 10 medical students and 2 physicians. The medical students were in their penultimate or final year of study, while the 2 physicians included 1 foundation physician and 1 honorary research clinical fellow. All participants demonstrated an interest in endocrinology, having been active members of the CoMICs initiative for at least 1 year and engaged in ongoing development of the CoMICs guideline. Their participation reflected both their exploratory career interests and commitment to evidence-based knowledge dissemination.

Semistructured interviews explored participants’ experiences with clinical guidelines, perceptions of the CoMICs format, and the personal or professional impact of their involvement. Students were also asked to reflect on how the experience shaped their views on endocrinology, academic collaboration, and the role of creative dissemination in medicine. The interview guide was aligned with the 2 objectives of the study and focused on clarity, usability, coproduction dynamics, and suggestions for future improvement. All interviews were audio-recorded and transcribed verbatim. Saturation was considered reached when participants could no longer provide additional insights or perspectives relevant to the research questions.

### Ethical Considerations

The study was reviewed and received ethics approval from the Science, Technology, Engineering, and Mathematics Committee at the University of Birmingham (ERN_2965-Aug2024). All data were anonymized to safeguard the participants’ information. Written informed consent was obtained from all participants before the interview, in accordance with institutional guidance and the approved ethics protocol. Participation was voluntary, and participants were not compensated for their time.

### Data Analysis

Interview data were analyzed using reflexive thematic analysis following the six-phase framework by Braun and Clarke [10]:

1. Familiarization: transcripts were reviewed in depth, with initial impressions documented.
2. Generating initial codes: RS undertook repeated familiarization with the transcripts before manually coding meaningful segments of text. Initial codes were developed inductively and generated directly from the data at a semantic level. Coding was data-driven and iterative, with reflexive notes maintained throughout to document analytic decisions and support ongoing refinement. Preliminary codes were shared with the research team, and team discussions informed the refinement and consolidation of the initial coding set.
3. Constructing initial themes: related codes were clustered to form preliminary candidate themes.
4. Reviewing themes: themes were refined for coherence and distinctiveness, with weak or overlapping themes revised or removed.
5. Defining and naming themes: final themes were clearly defined, named, and supported with representative quotations through group discussion. Generative artificial intelligence assistance was used only to refine subthemes, and all analytic decisions were confirmed through team consensus.
6. Producing the report: themes were organized in relation to the study objectives and integrated with relevant literature. This approach enabled a nuanced understanding of the practical and pedagogical value of audiovisual dissemination and provided insight into how cocreation experiences can foster medical students’ interest in specialty areas and academic pathways. The themes were contextualized using findings from the literature review, highlighting the potential for CoMICs to address longstanding dissemination challenges while also serving as a platform for early academic engagement.

## Results

### Overview

The 4-part guideline CoMICs series for health care professionals has accumulated 1218 views on YouTube and 1331 views on X (as of November 22, 2025) since its creation. The patient and public versions were created in English, Bengali, Serbian, Tamil, Greek, Georgian, and Brazilian Portuguese and have been viewed 1884 times on YouTube and 2420 times on X (as of November 22, 2025) since their creation. The final CoMICs videos are publicly accessible via YouTube (Multimedia Appendix 1).

### Thematic Analysis of Interviews

#### Overview

A total of 15 participants—12 (80%) medical students and 3 (20%) senior health care professionals—involved in the development or review of the GIAI CoMICs series were interviewed. Thematic analysis revealed 5 overarching themes with associated subthemes and codes (Table 1), with supporting illustrative quotes.

**Table 1.** Thematic analysis results.

Theme and subtheme		Code
Accessibility and usability		Use NICE^a^ for guidelines Written guidelines are too long Prefer short videos Guidelines difficult to navigate in clinical settings Multilingual versions broaden access Supports diverse learner backgrounds
Visual and cognitive engagement		
Quick refreshers		Use videos as refreshers before clinics Videos used during breaks or on commutes
Clinician use cases		Visuals improve learning and retention Animations clarify physiology and treatments
Credibility and trust		Expert review adds legitimacy Professional tone builds trust Referencing and consistency enhance credibility
Empowerment through cocreation		
Workflow friction		Students gained confidence and communication skills Challenges in feedback and team coordination Improved time and project management skills
Inclusivity and cultural reach		Multilingual versions increase reach Non-English speakers benefit from translations Can be shared with patients from diverse backgrounds

a NICE: National Institute for Health and Care Excellence.

#### Accessibility and Usability

Participants reported that traditional guidelines were often inaccessible in time-constrained settings, particularly during clinical work or exam preparation. Students and clinicians alike praised CoMICs as practical alternatives that distilled core messages into short, visually engaging formats. Furthermore, participants highlighted that multilingual CoMICs videos broadened accessibility and usability for parents and learners from diverse linguistic backgrounds, enhancing inclusivity and reach, especially where written English guidelines pose barriers:

*Even just a quick 2-minute summary video is more useful than scrolling through 30 pages.*

[Medical student]

#### Visual and Cognitive Engagement

CoMICs’ design—combining narration, visual metaphors, and sequential storytelling—was credited with enhancing comprehension and retention. Students appreciated the ability to review content quickly and repeatedly, while health care professionals used the videos as just-in-time learning tools before clinical duties:

*The animations helped me understand the physiology without flipping through the textbook.*

[CoMICs student team member]

#### Credibility and Trust

The rigorous review process, which involved guideline authors and clinical experts, was critical to establishing credibility. Students valued knowing their contributions would be clinically validated, while reviewers endorsed the professional standard of the final product:

*If you know the guideline authors reviewed it, it feels legitimate—not just a student project.*

[Medical student]

#### Empowerment Through Cocreation

Student participants expressed a strong sense of empowerment, seeing their work translated into tangible educational resources. They highlighted growth in communication, critical thinking, and teamwork skills. The experience also offered early exposure to academic endocrinology and knowledge translation, reinforcing interest in future academic careers:

*I never thought a video I helped make would be used by actual doctors.*

[Medical student]

Despite some communication delays and revision bottlenecks, students viewed the challenges as valuable professional learning opportunities.

#### Inclusivity and Cultural Reach

Multilingual adaptations were particularly valued for expanding access. Students noted that having videos in multiple languages allowed them to share resources with patients and peers from diverse backgrounds, enhancing equity in education and patient engagement:

*The Bangla version is great—I could share it with patients too.*

[Medical student]

This inclusive element positioned CoMICs as a globally relevant tool that could strengthen both practitioner education and patient-facing communication.

## Discussion

### Principal Findings

This study represents a novel contribution to the field of clinical guideline dissemination and medical education. To the best of our knowledge, it is among the first to explore the cocreation of audiovisual, narrative-based clinical resources—CoMICs—that are not only grounded in evidence-based guidelines but also designed for multilingual and multicultural relevance. Unlike previous CoMICs initiatives that focused on broad health topics and social media engagement, this study uniquely evaluates the transformation of a single clinical guideline into structured audiovisual outputs and examines the educational impact of the cocreation process on medical students. The visual structure of CoMICs supports the cognitive load theory by minimizing extraneous load and enabling learners to process guideline content more efficiently [11]. Previous reviews have emphasized the limited effectiveness of traditional dissemination methods (eg, printed guidelines and lectures) unless paired with contextual relevance, interactivity, and credible authorship [5,7,12,13]. CoMICs address these limitations through visual storytelling, coproduction with guideline authors, and multilingual delivery, making them a more adaptable and engaging format for real-world use. This aligns with emerging innovative dissemination practices, which emphasize concise, visually driven formats to improve the reach and uptake of health evidence [14].

Furthermore, this study supports growing calls in the health care education literature for multifaceted, user-driven dissemination models [15]. Notably, CoMICs serve not only as tools to improve practitioner guideline uptake but also as platforms for early-career professional development. Medical students involved in the production process gained firsthand experience in academic endocrinology, communication, interprofessional collaboration, and health education. Such active involvement can foster future interest in both clinical specialties and research careers—an impact rarely explored in the dissemination literature.

Our findings can be contextualized within dissemination science frameworks. The Framework for Knowledge Transfer by Lavis et al [16] proposes that effective dissemination depends on 5 core questions: what should be transferred, to whom, by whom, how, and with what effect. CoMICs directly addresses each of these domains: it condenses evidence-based guideline messages (what), targets medical students and patients (to whom), involves credible experts and peers (by whom), delivers content through short multilingual videos and social media (how), and measures engagement and understanding (with what effect). By mapping CoMICs to these principles, our approach provides a structured, theoretically grounded model for disseminating evidence through digital media.

The strength of this study lies in its dual-impact design, which simultaneously addresses the challenges of guideline dissemination and supports the development of future academic clinicians. The cocreative model provided students with meaningful, mentored engagement in real-world implementation efforts, resulting in a finished product with practical utility for health care professionals and patients alike. The inclusion of multilingual and culturally relevant content further extends CoMICs’ global reach, especially in resource-limited or linguistically diverse health care settings. Additionally, by capturing reflections from both clinical experts and student collaborators, the study offers a rich, balanced qualitative perspective on how innovation, collaboration, and mentorship can enhance both educational quality and clinical relevance.

### Limitations

We did not measure actual changes in clinical behavior or patient outcomes. While the findings indicate strong potential, objective evidence of impact remains limited. Moreover, because the interviewees were directly involved in developing the resources, positive bias may have influenced responses, potentially limiting the generalizability of the findings. It is also important to note that quantitative metrics, such as video views and engagement figures, were included solely to contextualize the qualitative findings, as the primary aim of this study was to explore the co-design experience rather than to evaluate dissemination outcomes. Furthermore, evaluating the broader impact of CoMICs on students and patients who viewed the content was beyond the scope of this work. Future research [1-7] should focus on guideline adherence, decision-making confidence, and quality of patient care. Several user-generated insights, such as using the videos as “quick refreshers before clinics” or “during breaks or commutes,” suggest practical pathways for future implementation. These patterns indicate that CoMICs could be integrated into just-in-time learning strategies, on-shift clinical preparation, and student revision routines. Future work should examine how such informal use cases can be systematically incorporated into guideline uptake workflows and clinical education pathways.

### Conclusions

CoMICs offer a practical and engaging solution to barriers in guideline dissemination, improving accessibility and relevance through visual storytelling and multilingual adaptation. Importantly, involving medical students in their creation fosters early exposure to academic endocrinology and professional skill development, supporting interest in research and specialty careers. Future work should focus on evaluating clinical outcomes, cost-effectiveness, and integration into wider implementation systems, ensuring scalability across specialties and settings.

## Supplementary material

10.2196/83711 Multimedia Appendix 1 YouTube hyperlinks to the 4-part glucocorticoid-induced adrenal insufficiency guideline Concise Medical Information Cines series and the 7 multilingual patient versions.

## Abbreviations

CoMICs: Concise Medical Information Cines
GIAI: glucocorticoid-induced adrenal insufficiency
