# Supplementary material for: Enhancing Engagement With Endocrine Guidelines and Fostering Medical Student Interest Through Concise Medical Information Cines: Qualitative Co-Design Study
Source: JMIR Med Educ. 2026 Jan 27;12:e83711. doi: 10.2196/83711 (PMC12844835; doi:10.2196/83711)
Supplement: Multimedia Appendix 1 [file mededu-v12-e83711-s001.docx]

**SUPPLEMENTARY 1 – Links to YouTube CoMIC videos**

ESE and ES Clinical Practice guideline: Glucocorticoid-induced Adrenal Insufficiency – part ¼

| **Name of YouTube Video** | **URL** |
| --- | --- |
| ESE and ES Clinical Practice guideline: Glucocorticoid-induced Adrenal Insufficiency – part 1/4 | <https://youtu.be/zcXHvi0Imiw?si=Nvbrh8SZgPE1wCuG> |
| ESE and ES Clinical Practice guideline: Glucocorticoid-induced Adrenal Insufficiency – part 2/4 | <https://youtu.be/rTfPdy5v3zU?si=fEofZ_d2Lxvzd0Js> |
| ESE and ES Clinical Practice guideline: Glucocorticoid-induced Adrenal Insufficiency – part 3/4 | <https://youtu.be/O2FRwMCFlUs?si=MwNt22-dfH4pG9AE> |
| ESE and ES Clinical Practice guideline: Glucocorticoid-induced Adrenal Insufficiency – part 4/4 | <https://youtu.be/AZskdmlonSA?si=uZg_cJ4_V9QegVjS> |
| CoMICsLite Episode 50: Glucocorticoid induced adrenal insufficiency – Info in English | <https://youtu.be/57J4OMIOH9g?si=B03dMGwVDsK0Lel_> |
| CoMICsLite Episode 52: Glucocorticoid induced adrenal insufficiency – Info in Bengali | <https://youtu.be/rJddotKtivk?si=1VTfKAjHH4ATumdZ> |
| CoMICsLite Episode 51: Glucocorticoid induced adrenal insufficiency – Info in Serbian | <https://youtu.be/ITDH0xcGUIs?si=0u6xX8TcsgpqbFOX> |
| CoMICsLite Episode 90: Glucocorticoid induced adrenal insufficiency – Info in Brazil Portugese | <https://youtu.be/-8oLzn4MmCM?si=R8MJzEAPDlDfOWu0> |
| CoMICsLite Episode 94: Glucocorticoid induced adrenal insufficiency – Info in Tamil | <https://youtu.be/KvFulCWkKgo?si=9Ke_R3NWJALynejD> |
| CoMICsLite Episode 98: Glucocorticoid induced adrenal insufficiency – Info in Greek | <https://youtu.be/D1ZQFWZ_uyU?si=67hUka5z5ToY-BLa> |
| CoMICsLite Episode 128: Glucocorticoid induced adrenal insufficiency – Info in Georgian | <https://youtu.be/UFlXOWcsxrA?si=BCH2MxBq9-MaAdO6> |
